# Physical activity and survival in chronic comorbidity among adult HIV patients in Ethiopia: a prospective cohort study

**DOI:** 10.1186/s12879-023-08651-9

**Published:** 2023-10-07

**Authors:** Yadessa Tegene, Selamawit Mengesha, Andargachew Kassa, Alemayehu Toma, Mark Spigt

**Affiliations:** 1 School of Public Health, College of Medicine and Health Science, Hawassa University, Hawassa, Ethiopia; 2 School of Nursing, College of Medicine and Health Science, Hawassa University, Hawassa, Ethiopia; 3 School of Medicine, College of Medicine and Health Science, Hawassa University, Hawassa, Ethiopia; 4 School CAPHRI, Department of Family Medicine, Maastricht University, Maastricht, Netherlands; 5 General Practice Research Unit, Department of Community Medicine, UiT the Arctic University of Norway, Tromsø, Norway

**Keywords:** Chronic comorbidity, Incidence rate, People living with HIV, Physical activity, Ethiopia

## Abstract

**Background:**

Antiretroviral therapy enables people living with HIV to live long lives, and these advances have transformed HIV infection from an acute to a chronic disease. Many non-communicable diseases, including type 2 diabetes, heart disease, and stroke, are influenced by physical inactivity. Therefore, the aim of this study was to assess the level of physical activity and survival in chronic co-morbidity among adult people living with HIV in Ethiopia.

**Methods:**

An institution-based prospective cohort study of adult people living with HIV was conducted between 2019 and 2021. We included 422 people living with HIV at baseline. After the baseline visit, 364 patients without hypertension or diabetes, were followed up for two years. Nine trained nurses used a pre-tested, structured questionnaire to collect data during routine care consultations in three hospitals in southern Ethiopia. STATA version 15 was used to analyze the data. To estimate the survival probability of developing chronic comorbidities, a Kaplan-Meier survival curve was used. A Cox proportional hazards model was fitted to identify the predictors of the development of chronic comorbidities.

**Result:**

In the current study, 39% of the participants were found to have a low level of physical activity. Those who had self-management skills to maintain physical activity (*p* = 0.023), considered physical activity an important aspect of their HIV management (*p* = 0.003), and regularly attended social support groups (*p* = 0.002) had significantly higher levels of physical activity. The risk of chronic comorbidity increased over time, with a rate of 10.83 chronic comorbidities per 1000 persons per month. Lack of regular exercise [AHR: 2.04; 95% CI: (1.03, 5.13)], low physical activity [AHR: 2.01; 95% CI: (1.03, 7.89)], BMI greater than 25 kg/m^2^ [AHR: 2.74; 95% CI: (1.31, 5.12)] and low fruit and vegetable intake [AHR = 2.57; 95% CI: (1.28, 6.49)] were all associated with the development of chronic comorbidity.

**Conclusion:**

The prevalence of physical inactivity is high in the study population. A physical activity program for people living with HIV should be considered, and the promotion of self-management skills should be integrated into HIV care programs.

## Introduction

At the end of 2019, there were 38.0 million [36.2 million adults] people living with HIV (PLWH) worldwide, with 62% of them having access to life-saving Antiretroviral Therapy (ART) [1, 2]. With more than 17.7 million PLWH in Eastern Africa, it is the second most affected region in Africa, after South Africa [3, 4]. In 2016, the estimated prevalence of HIV in Ethiopia was 1.1% [5]. PLWHs who receive ART can live long and healthy lives, and this advancement has transformed HIV infection from an acute to a chronic disease [6, 7]. However, the toxic side effects of ART, long-term HIV infection, increased obesity, and visceral adiposity have made PLWHs more vulnerable to chronic non-communicable diseases (NCDs), such as cardiovascular disease and diabetes mellitus [8–10]. The presence of NCD comorbidities among PLWHs may impair HIV prognosis [11].

PLWH share many NCD risk factors, such as unhealthy diets (high intake of fat, salt, and refined sugars), physical inactivity, tobacco use, and harmful alcohol use [12, 13]. A systematic review of PLWHs from high-income countries found that physical activity improves health and functional capacity [14]. According to other systematic reviews and meta-analyses, physical activity is beneficial for adults with HIV in terms of cardiorespiratory fitness, strength, body composition, and quality of life [15]. A study conducted in the United States indicates the benefit of PA in decreasing comorbidities in PLWHs [16]. The World Health Organization (WHO) guidelines on PA for health recommend 150 min of moderate intensity or 75 min of vigorous intensity per week for adults aged 18–64 for substantial health benefits [17]. Given the substantial benefits of regular PA, cross-sectional studies conducted in low-income countries indicate that most PLWHs are insufficiently physically active [18, 19].

An unhealthy diet is one of the most important modifiable risk factors for the most common NCD, as it can contribute to the development of conditions such as hypertension and type 2 diabetes mellitus (T2DM), as well as being overweight or obese, which are both risk factors for many NCDs [20]. There is growing concern in the public health sector that a poor diet increases the risk of chronic diseases and nutrition problems [21]. Unhealthy diets, particularly a lack of fruits and vegetables, are also major behavioral risk factors for NCDs [22]. Despite this, consumption of these foods remains significantly lower than the recommended level [22]. Several studies have found that the proportion of PLWHs who are overweight or obese is increasing in Sub-Saharan Africa [23]. The World Health Organization has issued warnings predicting the emergence of NCDs in resource-limited countries in the coming decades due to an increase in risk factors such as being overweight or obese [23]. In Africa, the impact of HIV/AIDS on food insecurity is well documented [24]. According to research, food insecurity and less diversified food are linked to a number of key metabolic risk factors, including obesity, overweight, and dyslipidemia [25, 26].

Despite a few cross-sectional studies conducted in Ethiopia, there is insufficient evidence on PA level and survival in chronic comorbidity among HIV patients. Such research could be critical in identifying and differentiating modifiable factors that are certain to reduce chronic comorbidity in these subjects. Therefore, the purpose of this study was to assess PA and survival in chronic comorbidity among adult PLWHs in Ethiopia. The results of this study could be used to develop strategies to reduce the risks associated with chronic comorbidities in adult HIV patients.

## Methods

### Study design, setting, participants and sampling

An institution-based prospective follow-up study was conducted from May 2019 to April 2021 in three selected hospitals: one comprehensive specialized hospital and two general hospitals. Hawassa University Comprehensive Specialized Hospital (HUCSH), a tertiary-level hospital, delivers specialized and referral services for general hospitals. The two general hospitals, Adare and Yirgalem, deliver secondary-level health care, providing preventive and curative services that require diagnostic facilities and therapeutic intervention [27]. HUCSH and Adare general hospitals are located in Hawassa town, the capital of the Sidama regional state and the South Nation Nationality Peoples Region (SNNPR) of Ethiopia, which is located 275 km south of Addis Ababa, the capital of Ethiopia. HUCSH, Adare, and Yirgalem general hospitals at the beginning of this study gave ART service to 2553, 1821, and 1476 HIV patients, respectively.

The study had two rounds of follow-up over a 24-month period. For this analysis, we used data from both the baseline and the one and two year follow-up measurements. The study sample was selected from adult PLWHs (18 years plus) enrolled in ART care and visiting the three selected hospitals during the study period. The study excluded pregnant and lactating women. The sample size required was calculated using the statistical software Epi Info, version 7, under the following assumptions: With a 95% confidence interval, 80% power, and a 1:1 exposed-to-non-exposed ratio, with a 10% non-response rate, the final sample size was 422 [28]. Proportional allocation was used to determine the number of study units to be sampled from each facility. Based on this, 184, 131, and 107 study participants were selected from HUCSH, Adare, and Yirgalem general hospitals, respectively. Participants in the study were chosen at random from the ART clinic. Baseline data from the same study population were used to assess the physical activity levels and associated factors of our study participants, (https://bmcinfectdis.biomedcentral.com/articles/10.1186/s12879-022-07120-z).

### Data collection methods and procedures

Data was collected by nine trained nurses during routine consultations using an interviewer-administered questionnaire. To collect data on the participants’ level of PA, we used the short form of the International Physical Activity Questionnaire (IPAQ) [29]. We substituted alternative physical activities with nearly the same Metabolic Equivalent of Task (MET) because several of the examples of PA were not regular activities in Ethiopia [30]. Fast biking was replaced by rope jumping under the category of vigorous PA. Bicycling at a steady speed and double tennis were replaced with cleaning and gardening in the category of moderate PA. The questionnaire was translated to Amharic after the cultural adaptation and then retranslated to the original version to ensure consistency. PA data was given as a continuous score using MET-min per week (MET level x minutes of activity x events per week) or as a categorical variable with three levels: low, moderate, and high. Low active or inactive participants were defined as those who did not meet the moderate or high requirement.

Participants who fulfilled one of the following criteria were categorized in the moderate group.

- Performing at least 20 min of vigorous activity on three or more days a week or;
- Performing moderate-intensity activity or walking for at least 30 min on five or more days a week or;
- Any combination of moderate-intensity activity, vigorous activity or walking on five or more days achieving at least 600 MET-min per week.

Participants were categorized into the high activity group, if they fulfilled one of the following criteria:

- Performing vigorous-intensity activity on a minimum of three days a week and achieving at least 1500 MET-min per week or;
- Any combination of moderate-intensity activity, vigorous activity or walking on seven days achieving at least 3000 MET-min per week.

Data for the Household Dietary Diversity Score (HDDS) [31] and Household Food Insecurity Access Scale (HFIAS) [32] were collected using the Food and Nutrition Technical Assistance (FANTA) indicator guide. Anthropometric measurements, such as height, were taken with a stadiometer (Seca Germany), which was used to position the patient in the Frankfert plane and record to the nearest 0.1 cm. A pretested and calibrated digital Seca® scale was used to measure weight to the nearest 0.1 kg. The BMI was calculated as the weight in kilograms divided by the height in meters squared. According to the WHO classification, normal weight was defined as a BMI of 18.5 to 24.9 kg/m^2^, overweight as a BMI of 25 to 30 kg/m^2^, and obesity as a BMI of more than 30 kg/m^2^ [33].

By consistently measuring the left arm three times at a 5-minute interval, blood pressure (BP) was measured with a standard mercury sphygmomanometer BP cuff with the proper cuff size. The average of the two most recent readings was taken, and high blood pressure (hypertension) was diagnosed using WHO criteria of systolic BP ≥ 140 mmHg or diastolic BP ≥ 90 mmHg [34]. Random blood glucose levels were determined by using the Fia Biomed Blood Glucose Meter (Glucometer) Salut by finger puncture. According to the American Diabetes Association’s guidelines, fasting plasma glucose levels ≥ 126 mg/dL, 2 h plasma glucose ≥ 200 mg/dL during an oral glucose tolerance test, hemoglobin A1C ≥ 6.5% and in a patient with classic symptoms of hyperglycemia or hyperglycemic crisis, a random plasma glucose ≥ 200 mg/dL are defined as diabetes [35]. Participants were classified as having chronic comorbidity if they had either diabetes or hypertension, or both. Participants who were unaware of the fact that they had diabetes and/or hypertension were linked to the respective hospitals for further diagnosis and management of their conditions.

Self-management in HIV patients was assessed using the HIV Self-management Scale [36, 37]. The 20 items on the HIV Self-Management Scale are divided into three domains. The domains include 12 questions about daily health practices for managing HIV, 3 questions about social support for managing HIV, such as “Attending support groups is an important part of my HIV self-management strategy,“ and 5 questions about the chronic nature of managing HIV (e.g., “I have accepted that HIV is a life-long condition that can be managed”). Each item is scored on a 0–3 scale: 0 = not applicable, 1 = none of the time, 2 = some of the time, and 3 = all of the time. Each domain is scored separately and divided by the number of items in that domain and the total score of the scale was calculated by summing items in all domains and the possible score thus ranged from 0 to 60. Out of the 20 items on the HIV self-management scale, for our current study, we have used five items that we deemed could be related to physical activity practice.

### Data processing and analysis

Data were analyzed using STATA version 15. Descriptive statistics were presented in the form of frequency, percentage, mean and standard deviation to describe the sample’s baseline sociodemographic characteristics and other variables. The survival probabilities of developing chronic comorbidities (hypertension and diabetes mellitus) were estimated using a Kaplan-Meier (KM) survival curve, and the probability of survival curves between various groups were compared using Log rank testing. To find predictors of time to chronic comorbidity, bivariable and multivariable Cox proportional hazards models were used. For further analysis, independent variables with a *P*-value of ≤ 0.25 in the bivariable analysis were incorporated into the final multivariable model. The Schoenfeld residual global test and the log-Log plot test were used to assess the assumptions of the Cox proportional hazard regression model. The strength of the association and statistical significance were assessed using an Adjusted Hazard Ratio (AHR) with 95% confidence intervals (CI).

## Results

### Socio-demographic characteristics of the study participants

A total of 364 patients were prospectively followed. The mean age of the study participants was 40.9 ± 9 years. The majority of the study’s participants were women (62%), single (49%), orthodox Christians (50%), and between the ages of 31 and 40 (42%). Most of them lived in urban areas (93%), had completed secondary education (36%), and were self-employed (30%). A little more than half of the study participants had low incomes (51%), (Table 1).

**Table 1:** Socio-demographic characteristics of the study participants, South Ethiopia, May 2019-June 2021, (*n* = 364)

Variable	Frequency	Percent
Mean (SD) age 40.9 ± 9		
Sex		
Male	139	38
Female	225	62
Age		
< 20	4	1
21–30	33	9
31–40	154	42
41–50	113	31
51–60	60	17
Marital status		
Single	177	49
Married	52	14
Divorced	62	17
Widowed	73	20
Religion		
Orthodox	180	50
Protestant	123	34
Muslim	55	15
Others	6	2
Level of education		
No formal education	39	11
Primary education	95	26
Secondary education	132	36
Tertiary education	98	27
Occupation		
Government employee	79	22
Private employee	108	30
Daily-laborer	42	12
Student	46	13
Merchant	56	15
Others	33	9
Place of residence		
Urban	340	93
Rural	24	7
Income level in ETB		
< 1500	184	51
≥ 1500	180	49

Private Employee: A person who works for a private employer or in private organization and receives regular remuneration in salary

1 USD=29.21 Ethiopian Birr, 2019. Other religions: Catholic and Adventist. Other occupations: farmers and house wife

### Clinical, anthropometric, dietary and other health related characteristics of the study participants

The majority of the patients (94%) had been using ART for at least 24 months, had BMI ≤ 25 kg/m^2^ (77%), and had no recent history of anemia (81%). A little more than half of the study participants (55%) reported eating fruits and vegetables, but did not regularly engage in exercise (53%). Nearly all of them (99%) had never smoked cigarettes, and the majority (89%) did not currently consume alcohol (Table 2).

**Table 2:** Clinical, anthropometric, dietary and other health related characteristics of the study participants, South Ethiopia, May 2019-June 2021, (*n* = 364)

Variable	Frequency	Percent
Duration of ART in months		
< 24	22	6
≥ 24	342	94
Recent anaemia		
Yes	68	19
No	296	81
BMI		
< 25 kg/m^2^	280	77
25-30 kg/m^2^	64	18
> 30 kg/m^2^	18	5
Regular physical exercise		
Yes	172	47
No	192	53
Current alcohol consumption		
Yes	40	11
No	324	89
History of cigarette smoking		
Yes	4	1
No	360	99
Fruit and vegetable consumption		
Yes	200	55
No	164	45
HHFIS		
Secured	203	56
Insecure	161	44

*HHFIS *Household food insecurity scale. Anaemia: <120 g/L for non−pregnant women and, < 130 g/L for men

### Proportions of study participants engaging in vigorous, moderate and walking physical activity

Most participants, 53% of men and 48% of women, did not engage in vigorous PA (hard lifting, digging, aerobics, and rope jumping). Male participants (9%) were slightly more likely than female (7%) to have ≥ 150 min of vigorous PA per day. A slightly higher percentage of men (11%) than women (5%) participated in moderate PA (cleaning, gardening, and carrying light goods) per day. Only a small percentage of men and women (2%) did not walk per week. Neither one of them, however, walked for 150 min per day or more (Table 3).

**Table 3:** Proportion of adult HIV patients who are engaging in vigorous, moderate and walking PA, South Ethiopia, May 2019-June 2021, (*n* = 364)

Number of minutes per day	Physical activity type					
	Vigorous		Moderate		Walking	
	Male	Female	Male	Female	Male	Female
0	53	48	18	12	2	2
10–30	13	14	27	35	78	87
31–60	14	19	25	29	14	9
61–149	11	12	19	19	4	1
≥ 150	9	7	11	5	1	0
Total	100	100	100	100	100	100

### Physical activity level of the participants

Most participants (39%) had low PA levels on the categorical IPAQ scale. Compared to 32% of participants who lived in urban areas, 54% of those who lived in rural areas engaged in high levels of PA. Rural participants reported statistically higher PA (*p* = 0.048) and IPAQ scores (*p* = 0.032) compared to their urban counterparts (Table 4).

**Table 4:** Physical activity level measured by IPAQ among adult HIV patients, South Ethiopia, May 2019-June 2021, (*n* = 364)

Variable	Place of residence						
	**Urban**		**Rural**		**Total**		***P*** **value**
	**n**	**%**	**n**	**%**	**n**	**%**	
Physical activity level							0.048*
Low	134	39	6	25	140	39	
Moderate	104	31	5	21	109	30	
High	102	30	13	54	115	32	
	Median (IQR)		Median (IQR)		Median (IQR)		
Vigorous activity							
Days per week	2 (0–4)		1 (0-4.75)		2 (0–4)		0.96 †
Minutes per day	30 (0–60)		30 (0-120)		30 (0–60)		0.52 †
MET score	480 (0-2400)		480 (0-3840)		480 (0-2400)		0.48 †
Moderate activity							
Day per week	5 (3–5)		5(3–5)		5 (3–5)		0.40 †
Minute per day	60 (30–60)		60(30–120)		60 (30-112.5)		0.40 †
MET score	720 (420–1440)		1080 (630–2400)		820 (480–1620)		0.053
Walking activity							
Day per week	5 (3–5)		4 (3–5)		5 (3–5)		0.19 †
Minute per day	30 (30–30)		30 (22–30)		30 (30–30)		0.82 †
MET score	495 (297–495)		396 (222.75–495)		495 (297–495)		0.28 †
IPAQ total score	2475 (1095–4790)		3644 (2616–6347)		2605 (1095–5181)		**0.032 †**

Significance level, *p*<0.05.*χ2 test, †Mann−Whitney test. MET score: Metabolic Equivalent of Task,

^*IPAQ*International Physical Activity Questionnaire, *IQR*Inter Quartile Range^

### Physical activity level and self-management skill

In terms of study participants’ self-management skills, those who consistently reported success in maintaining PA (*P* = 0.023), saw PA as an important part of their HIV management plan (*P* = 0.003), and regularly participated in support groups (*P* = 0.002) had a significant association with a high level of PA (Table 5).

**Table 5:** Association between physical activity level and self-management skill of study participants, South Ethiopia, May 2019-June 2021, (*n* = 364)

Variable	Physical activity status						*P* value
	Low		Moderate		High		
	N	%	N	%	N	%	
Staying physically active (exercising) is an important part of my HIV management strategy							
Not applicable	10	42	8	33	6	24	0.003
None of the time	45	45	33	33	21	21	
Some of the time	42	50	18	21	24	29	
All of the time	43	27	50	32	64	41	
I have been successful at staying physically active (walking, exercising, stretching, weight lifting, physical work)							
Not applicable	14	48	7	24	8	28	0.023
None of the time	57	45	37	29	33	26	
Some of the time	44	43	29	28	30	29	
All of the time	25	24	36	34	44	42	
I have been attending support groups because I found that listening to someone’s testimony or personal story motivates me to take better care of myself							
Not applicable	29	43	20	29	19	28	0.002
None of the time	49	44	34	35	28	25	
Some of the time	30	45	24	36	13	19	
All of the time	32	27	31	26	55	47	
I have accepted that HIV is a chronic (or life-long) condition that can be managed							
Not applicable	18	44	11	27	12	29	0.91
None of the time	39	42	27	29	26	28	
Some of the time	22	34	19	29	23	36	
All of the time	61	37	52	31	54	32	

### Incidence of chronic comorbidity among the study participants

**Fig. 1:**
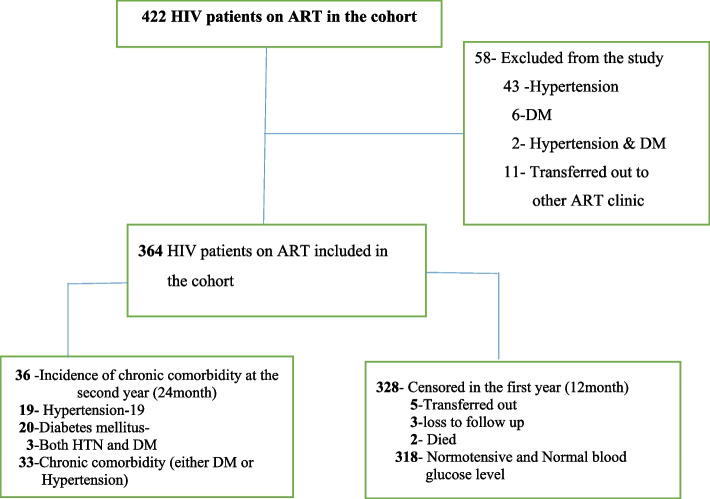
Flowchart diagram of the study profile of the participants in the three selected hospitals of south Ethiopia

During the follow-up period, 19 (5.2%) new hypertensive cases, 20 (5.5%) diabetes cases, and 3(0.8%) of them developed both DM and hypertension. The overall new cases of chronic comorbidity was 36 (10%), and the remaining 328 (90%) were censored. Of these censored patients, 318 (87%) were normotensive and had normal blood glucose levels; 5(1.4%) were transferred out to other ART clinics; 3 (0.8%) were lost to follow-up; and 2 (0.6%) were dead. The median survival time to develop chronic comorbidity was nine months, with an interquartile range IQR: 4.7–14.7 months. The incidence rate of chronic comorbidity was 10.83 per 1000 people per month with a total of 3224 patient-month observations (Fig. 1).

### Predictors of chronic comorbidity among the study participants

In the bivariate Cox regression model, household food insecurity, low fruits and vegetables, anemia, BMI, current alcohol intake, regular PA, and PA status all had *p*-values < 0.25. In the final multivariate Cox regression model, only four covariates, low fruits and vegetables, BMI, regular PA, and PA status, were statistically significant. PLWHs who had low fruit and vegetable consumption were three times more likely to develop chronic comorbidity than their counterparts [AHR = 2.57; 95% CI: (1.28, 6.49)]. When compared to those with a BMI of less than 25 kg/m^2^, those with a BMI greater than 25 kg/m^2^ had a three-fold [AHR = 2.74; 95% CI: (1.31, 5.12)] increased risk of developing chronic comorbidity. Patients who did not regularly exercise [(AHR = 2.04; 95% CI: (1.03, 5.13)] and those with poor levels of PA [(AHR = 2.01; 95% CI: (1.03, 7.89)] had a two-fold higher risk of developing chronic comorbidity than their counterparts (Table 6).

**Table 6:** Cox regression analysis of for predictors of chronic comorbidity among adult HIV patients attending ART clinic in three selected hospitals of South Ethiopia (*N* = 364)

Variables	Survival Status				CHR 95%CI	AHR 95%CI	*P*-value
	Event (%)	Censored (%)					
Food insecurity							
No	24	12	179	88	1	1	
Yes	12	8	149	92	2.40 (1.45, 3.97)	1.83 (0.95, 4.50	0.078
Low fruits and vegetables							
No	12	8	149	92	1	1	
Yes	9	5	155	95	1.89 (1.16, 3.12)	2.57 (1.28, 6.49)	0.010*
Anemia (< 11 g/dl)							
No	27	9.	269	91	1	1	
Yes	9	13	59	87	2.43 (1.38, 4.28)	0.46 (0.52, 2.83)	0.67
BMI							
≤ 25 kg/m2	19	7	261	93	1	1	
> 25 kg/m2	17	20	67	80	5.47 (3.18, 9.38)	2.74 (1.31, 5.12)	0.006*
Current alcohol consumption							
Yes	30	9	294	91	1	1	
No	6	15	34	85	1.58 (77, 3.25)	0.63 (0.52, 3.47)	0.63
Regular physical exercise							
Yes	26	15	146	85	1	1	
No	10	5	182	95	2.89 (1.72, 4.86)	2.04(1.03, 5.13)	0.041*
Physical activity status							
High	5	4	110	96	1	1	
Moderate	6	6	103	94		0.23 (0.34, 3.99)	0.82
Low	25	18	115	82		2.01 (1.03, 7.89)	0.045*

### Kaplan-meier survival analysis

**Fig. 2:**
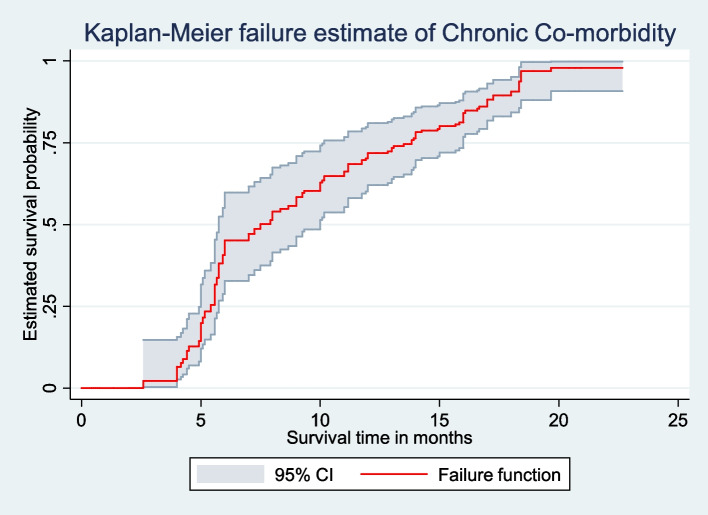
The Kaplan–Meier curve showing the survival probability of developing chronic comorbidity, South Ethiopia, (*n* = 364)

**Fig. 3:**
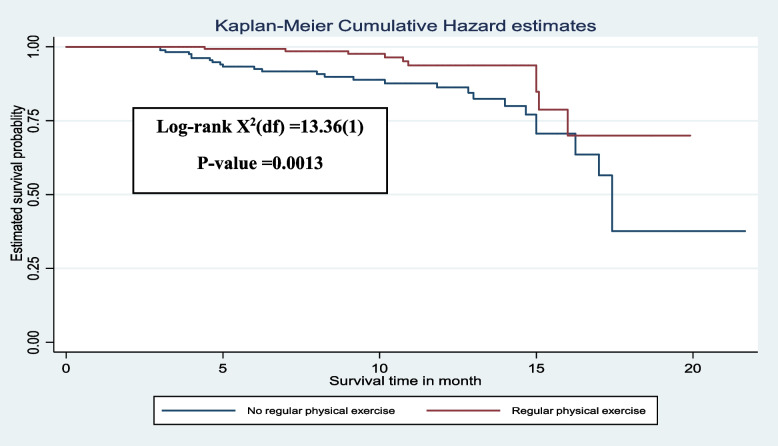
The Kaplan–Meier curve showing the survival probability of developing chronic comorbidity based on regular physical exercise, South Ethiopia, (*n* = 364)

**Fig. 4:**
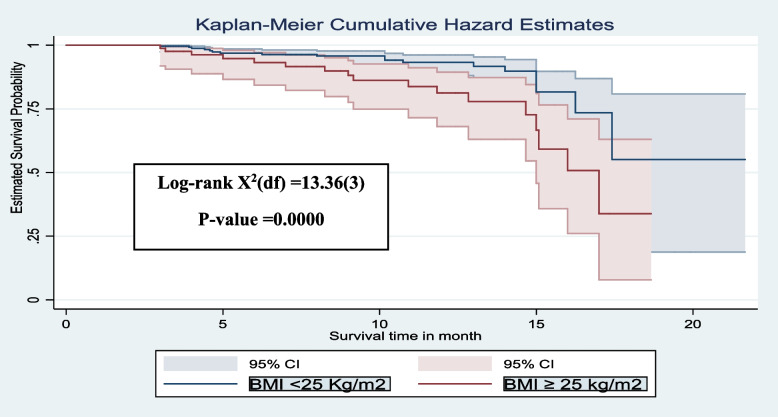
The Kaplan–Meier curve showing the survival probability of developing chronic comorbidity based on BMI, South Ethiopia, (*n* = 364)

The Kaplan-Meier survival curve revealed that among adult HIV patients, the risk of developing chronic comorbidity increased over time. Many of the cases were observed after 5 months of follow-up (Fig. 2). Additionally, patients who did not engage in regular exercise (Fig. 3), and those with a BMI of 25 kg/m^2^ and above (Fig. 4), had significantly higher estimated cumulative probabilities of developing chronic comorbidity over time.

## Discussion

The findings of this study show that most PLWHs had a low level of PA. Walking was the most frequently practiced PA. People living in rural areas were more physically active than those living in urban areas, yet sitting was also more common among the rural residents. Self-management skills of the participants was associated with a high level of PA. During the two-year follow-up period, 20 (5.5%) new cases of diabetes, 19 (5.2%) new cases of hypertension, and 3 new cases of both DM and hypertension were registered. The overall incidence rate of chronic comorbidity was 10.83 per 1000 people per month. A lack of regular physical exercise and a low level of PA, low fruit and vegetable consumption, and having a BMI greater than or equal to 25 kg/m^2^ were associated with the development of chronic comorbidity.

We found that the majority of participants (39%) had low levels of PA and slightly more than half (53%) of our study participants were not engaged in regular PA. This implies that they fell short of the 2020 Global Recommendations on PA for Health [38] recommended PA levels. Compared to previous studies on ART patients using the IPAQ system, the percentage of low PA level in our study is high [39, 40]. Similar to previous studies, walking was the most frequently practiced PA [41, 42]. Neither men nor women, however, walked for more than 150 min per week. Contrary to what we found, a research done among Saudi adults showed that 37.8% of men and 28.5% of women reported walking for 150 min or more every week [43]. Additionally, the Women’s Health Initiatives follow-up study showed that walking needs to be done for at least 30 min, five days a week, in order to protect against chronic illnesses like cardiac disease [44]. Therefore, promoting PA as an intervention strategy is crucial to preventing the added burden of chronic comorbidity among adult HIV patients.

In our study, 50% of participants from rural areas had high levels of PA, which is higher than that of urban dwellers. It is consistent with studies conducted in sub-Saharan African countries, Ethiopia, Northern Tanzania and Vietnam [19, 39, 45]. Most rural areas in Ethiopia lack access to transportation, thus people must travel large distances by foot for a variety of social reasons, such as moving from one farm site to another or moving from one location to another. Furthermore, rural residents frequently engage in manual labor and physical travel, which promotes robust and moderate PA, in contrast to their urban counterparts who appear to embrace sedentary habits [19]. On the other hand, sitting was also more common among the rural residents, and the average day per week of walking activity was also lower among the rural residents. A plausible reason for this could be that in the rural setup of the current study area, walking frequency could be influenced by the lack of pedestrian infrastructure such as sidewalks that may discourage people from regular walking. But the MET score obtained by walking activity among the rural residents was higher compared to their urban counterparts, and this reflects that rural dwellers walk long distances but not on a regular basis. As a result, healthcare professionals working in the ART clinic should promote awareness of the importance of regular PA among HIV patients.

The study participants’ self-management skills, such as those who saw maintaining PA as an important part of their HIV management strategy, attended social support groups, and those who had success in maintaining PA consistently, had a significant association with a high level of PA. There is supporting evidence from the study conducted in Thailand indicating self-management is an effective strategy for enhancing exercise behavior [46]. Therefore, health care providers working in ART clinics should pay attention to HIV patients’ self-management skills in order to increase PA levels and thereby decrease the risk of chronic comorbidity.

The lack of longitudinal studies on the incidence of chronic comorbidity in PLWHs in low-income settings is concerning. There is evidence from cross-sectional studies that the prevalence of chronic comorbidities (hypertension and diabetes mellitus) is high in the African population, including Ethiopia [47–49]. Our current study’s results revealed that among adult HIV patients, the hazard of developing chronic comorbidity increased over time, with an overall incidence rate of chronic comorbidities of 10.83 per 1000 person-months. The incidence rate of diabetes mellitus was 11 per 1000 people-year follow-up as an independent case, according to the retrospective cohort study done on PLWHs in Thailand [50]. There are more cases of hypertension, according to studies on the incidence in other parts of Ethiopia, Uganda, Tanzania, and South Africa [51–54]. The higher cut-off value used in this study to diagnose incident hypertension compared to some of the other studies may be contributing to the lower incidence. Incidence rate variations may also be caused by variations in sample size, study design (prospective vs. retrospective cohort), study area, and socio-demographic characteristics of study participants.

Our study found that patients who did not exercise regularly and those with low levels of PA had a considerably higher estimated cumulative probability of developing chronic comorbidity over time. PA has been shown in numerous studies to play a significant role in lowering the morbidity and mortality of various diseases. People of all ages can benefit from PA in terms of their physical health, psychological health, social health, and emotional health [55, 56]. In spite of the benefits that have been found, a sizable portion of PLWHs in sub-Saharan Africa, including Ethiopia, still do not exercise as part of their rehabilitation [57]. For these groups to have better health and experience a lower likelihood of developing chronic comorbidity, interventions that encourage PA will be crucial.

According to the results of the current study, people with a BMI of 25 kg/m^2^ or more had a significantly increased estimated cumulative likelihood of having a chronic comorbidity over time. In this study, being overweight is also another modifiable predictor of chronic comorbidity. There are similar studies showing that high BMI is a modifiable predictor of chronic comorbidity in PLWHs [28, 58, 59]. According to the study, which was conducted in two hospitals in the United Kingdom, the diagnosis of metabolic syndrome increased significantly as BMI increased [60]. Previous studies indicate the association between high BMI (obesity and overweight) and a lack of PA [61, 62]. For instance, a study conducted among adult PLWHs in South-west Ethiopia [63] shows that patients who had no regular PA were 1.3 times more likely to develop obesity/overweight compared to those who had. Therefore, it is crucial to support intervention programs that emphasize encouraging PA among HIV/AIDS patients.

Participants in the study who consumed less fruits and vegetables than their counterparts were also more likely to develop chronic comorbidity. This result is in line with a systemic review study that demonstrates the significant preventative potential of increasing vegetable and fruit consumption in relation to a range of diseases, including hypertension and diabetes mellitus [64]. WHO also emphasizes the inclusion of fruits and vegetables in our diets to promote health and lower the risk of certain non-communicable diseases (NCDs) [65]. Therefore, nutritional counselling intended to increase HIV patients’ consumption of fruits and vegetables should be taken into consideration to lower the chance of acquiring chronic comorbidity.

Our study has some limitations, such as the fact that it only focused on the incidence of hypertension and diabetes mellitus, two common chronic comorbidities in HIV patients. To better understand the incidence of chronic comorbidities in this particular population, long-term follow-up of chronic comorbidities such as liver disease, cancer, and respiratory disease should be considered. The majority of our study participants were younger; to be aware of the effect of age, a proportionate number of participants from an older group should also be taken into account. Self-reported information was used to obtain the data, which is subject to recall bias, social desirability bias, and interviewer bias. To minimize these biases, patients were given clear instructions about the benefits of the study. Although self-report measures are considered appropriate for large-scale surveys, further research may indicate the use of objective measures of physical activity, such as accelerometry, to support these findings.

## Conclusion

In conclusion, prevalence of physical inactivity among PLWHs is high. A high BMI, low intake of fruits and vegetables, low PA levels, a lack of regular physical exercise, self-management skill have been shown to significantly increase the risk of developing chronic comorbidity. Addressing modifiable risk factors such as encouraging PA, self-management skill and healthy diet should be integrated into HIV care.

## Data Availability

The datasets used and/or analyzed as part of this study are available from the corresponding author upon reasonable request.

## Abbreviations

AIDS: Acquired Immune Deficiency Virus
ART: Antiretroviral Therapy
AOR: Adjusted Odds Ratio
BMI: Body Mass Index
BP: Blood Pressure
CI: Confidence Interval
CVD: Cardiovascular Disease
HDDS: Household Dietary Diversity Score
HHFIAS: Household Food Insecurity Access Scale
HIV: Human Immunodeficiency Virus
HUCSH: Hawassa University Comprehensive Specialized Hospital
IDDS: Individual Dietary Diversity Score
NRTI: Nucleotide Reverse Transcriptase Inhibitors
NCD: Non-communicable diseases
OR: Odds Ratio
PI: Protein Inhibitors
PLWH: People Living with HIV
SPSS: Statistical Package for The Social Sciences
WHO: World Health Organization
